# Supplementary material for: Assessing Affective State in Laboratory Rodents to Promote Animal Welfare—What Is the Progress in Applied Refinement Research?
Source: Animals (Basel). 2019 Nov 25;9(12):1026. doi: 10.3390/ani9121026 (PMC6941082; doi:10.3390/ani9121026)
Supplement: Supplementary file 1 [file animals-09-01026-s001.pdf]

Assessing affective state in laboratory rodents to promote animal welfare - What is the progress in applied refinement research?

Paulin Jirkof [1], Juliane Rudeck [2], Lars Lewejohann [2,3]

[1] Department Animal Welfare and 3R, University of Zurich, Zurich, Switzerland

[2] German Federal Institute for Risk Assessment (BfR), German Center for the Protection of Laboratory Animals (Bf3R), Berlin, Germany

[3] Institute of Animal Welfare, Animal Behavior and Laboratory Animal Science, Freie University Berlin, Berlin, Germany

Supplement

S1: Reference list of literature search

a) Caron, A.; Lelong, C.; Bartels, T.; Dorchies, O.; Gury, T.; Chalier, C.; Benning, V. Clinical and anatomic pathology effects of serial blood sampling in rat toxicology studies, using conventional or microsampling methods. Regulatory Toxicology and Pharmacology 2015, 72, 429-439, doi:10.1016/j.yrtph.2015.05.022.

b) Bodden, C.; Siestrup, S.; Palme, R.; Kaiser, S.; Sachser, N.; Richter, S.H. Evidence-based severity assessment: Impact of repeated versus single open-field testing on welfare in C57BL/6J mice. Behavioural brain research 2018, 336, 261-268, doi:10.1016/j.bbr.2017.08.029.

c) George, R.P.; Barker, T.H.; Lymn, K.A.; Bigatton, D.A.; Howarth, G.S.; Whittaker, A.L. A Judgement Bias Test to Assess Affective State and Potential Therapeutics in a Rat Model of Chemotherapy-Induced Mucositis. Scientific reports 2018, 8, doi:10.1038/s41598-018-26403-7.

d) Hager, C.; Keubler, L.M.; Talbot, S.R.; Biernot, S.; Weegh, N.; Buchheister, S.; Buettner, M.; Glage, S.; Bleich, A. Running in the wheel: Defining individual severity levels in mice. PLoS biology 2018, 16, e2006159, doi:10.1371/journal.pbio.2006159.

e) Koch, A.; Gulani, J.; King, G.; Hieber, K.; Chappell, M.; Ossetrova, N. Establishment of Early Endpoints in Mouse Total-Body Irradiation Model. PloS one 2016, 11, e0161079, doi:10.1371/journal.pone.0161079.

f) Miller, A.; Burson, H.; Soling, A.; Roughan, J. Welfare Assessment following Heterotopic or Orthotopic Inoculation of Bladder Cancer in C57BL/6 Mice. PloS one 2016, 11, e0158390, doi:10.1371/journal.pone.0158390.

g) Pfeiffenberger, U.; Yau, T.; Fink, D.; Tichy, A.; Palme, R.; Egerbacher, M.; Rulicke, T. Assessment and refinement of intra-bone marrow transplantation in mice. Lab Anim 2015, 49, 121-131, doi:10.1177/0023677214559627.

h) Roughan, J.V.; Coulter, C.A.; Flecknell, P.A.; Thomas, H.D.; Sufka, K.J. The Conditioned Place Preference Test for Assessing Welfare Consequences and Potential Refinements in a Mouse Bladder Cancer Model. PloS one 2014, 9, doi:10.1371/journal.pone.0103362.

i) Wheeler, R.R.; Swan, M.P.; Hickman, D.L. Effect of multilevel laboratory rat caging system on the well-being of the singly-housed Sprague Dawley rat. Lab Anim 2015, 49, 10-19, doi:10.1177/0023677214547404.

j) Whittaker, A.L.; Lymn, K.A.; Nicholson, A.; Howarth, G.S. The assessment of general well-being using spontaneous burrowing behaviour in a short-term model of chemotherapy-induced mucositis in the rat. Lab Anim 2015, 49, 30-39, doi:10.1177/0023677214546913.

k) Jirkof, P.; Tourvieille, A.; Cinelli, P.; Arras, M. Buprenorphine for pain relief in mice: repeated injections vs sustained-release depot formulation. Laboratory Animals 2015, 49, 177-187, doi:10.1177/0023677214562849.

l) Moller, C.; Wolf, F.; van Dijk, R.M.; Di Liberto, V.; Russmann, V.; Keck, M.; Palme, R.; Hellweg, R.; Gass, P.; Otzdorff, C., et al. Toward evidence-based severity assessment in rat models with repeated seizures: I. Electrical kindling. Epilepsia 2018, 59, 765-777, doi:10.1111/epi.14028.

m) Hohlbaum, K.; Bert, B.; Dietze, S.; Palme, R.; Fink, H.; Thone-Reineke, C. Systematic Assessment of Well-Being in Mice for Procedures Using General Anesthesia. Journal of visualized experiments : JoVE 2018, 10.3791/57046, doi:10.3791/57046.

n) Augustsson, H.; Lindberg, L.; Hoglund, A.U.; Dahlborn, K. Human-animal interactions and animal welfare in conventionally and pen-housed rats. Laboratory Animals 2002, 36, 271-281, doi:10.1258/002367702320162388.

o) Balcombe, J.P.; Barnard, N.D.; Sandusky, C. Laboratory routines cause animal stress. Contemp Top Lab Anim 2004, 43, 42-51.

p) Boissy, A.; Manteuffel, G.; Jensen, M.B.; Moe, R.O.; Spruijt, B.; Keeling, L.J.; Winckler, C.; Forkman, B.; Dimitrov, I.; Langbein, J., et al. Assessment of positive emotions in animals to improve their welfare. Physiol Behav 2007, 92, 375-397, doi:10.1016/j.physbeh.2007.02.003.

q) Spani, D.; Arras, M.; Konig, B.; Rulicke, T. Higher heart rate of laboratory mice housed individually vs in pairs. Laboratory Animals 2003, 37, 54-62, doi:Doi 10.1258/002367703762226692.

r) Van Loo, P.L.P.; Kuin, N.; Sommer, R.; Avsaroglu, H.; Pham, T.; Baumans, V. Impact of 'living apart together' on postoperative recovery of mice compared with social and

individual housing. Laboratory Animals 2007, 41, 441-455, doi:Doi 10.1258/002367707782314328.

s) Prior, H.; Marks, L.; Grant, C.; South, M. Incorporation of capillary microsampling into whole body plethysmography and modified Irwin safety pharmacology studies in rats. Regulatory toxicology and pharmacology : RTP 2015, 73, 19-26, doi:10.1016/j.yrtph.2015.06.002.

t) Morley-Fletcher, S.; Rea, M.; Maccari, S.; Laviola, G. Environmental enrichment during adolescence reverses the effects of prenatal stress on play behaviour and HPA axis reactivity in rats. The European journal of neuroscience 2003, 18, 3367-3374, doi:10.1111/j.1460-9568.2003.03070.x.

u) Klein, Z.A.; Padow, V.A.; Romeo, R.D. The effects of stress on play and home cage behaviors in adolescent male rats. Developmental psychobiology 2010, 52, 62-70, doi:10.1002/dev.20413.

v) Reinhold, A.S.; Sanguinetti-Scheck, J.I.; Hartmann, K.; Brecht, M. Behavioral and neural correlates of hide-and-seek in rats. Science (New York, N.Y.) 2019, 365, 1180-1183, doi:10.1126/science.aax4705.

w) Panksepp, J.; Burgdorf, J. "Laughing" rats and the evolutionary antecedents of human joy? Physiol Behav 2003, 79, 533-547, doi:10.1016/s0031-9384(03)00159-8.

x) Richter, S.H.; Kastner, N.; Kriwet, M.; Kaiser, S.; Sachser, N. Play matters: the surprising relationship between juvenile playfulness and anxiety in later life. Animal Behaviour 2016, 114, 261 - 271, doi:https://doi.org/10.1016/j.anbehav.2016.02.003.

y) Bobrovskaya, L.; Beard, D.; Bondarenko, E.; Beig, M.I.; Jobling, P.; Walker, F.R.; Day, T.A.; Nalivaiko, E. Does exposure to chronic stress influence blood pressure in rats? Auton Neurosci-Basic 2013, 177, 217-223, doi:10.1016/j.autneu.2013.05.001.

z) Burgdorf, J.; Panksepp, J.; Moskal, J.R. Frequency-modulated 50 kHz ultrasonic vocalizations: a tool for uncovering the molecular substrates of positive affect. Neuroscience and biobehavioral reviews 2011, 35, 1831-1836, doi:10.1016/j.neubiorev.2010.11.011.

aa) Colpaert, F.C.; Meert, T.; Dewitte, P.; Schmitt, P. Further Evidence Validating Adjuvant Arthritis as an Experimental-Model of Chronic Pain in the Rat. Life Sci 1982, 31, 67-75, doi:Doi 10.1016/0024-3205(82)90402-7.

bb) Dinh, H.K.; Larkin, A.; Gatlin, L.; Piepmeier, E. Rat ultrasound model for measuring pain resulting from intramuscularly injected antimicrobials. Pda J Pharm Sci Tech 1999, 53, 40-43.

cc) Jourdan, D.; Ardid, D.; Chapuy, E.; Eschalier, A.; Le Bars, D. Audible and ultrasonic vocalization elicited by single electrical nociceptive stimuli to the tail in the rat. Pain 1995, 63, 237-249, doi:10.1016/0304-3959(95)00049-x.

dd) Jourdan, D.; Ardid, D.; Eschalier, A. Analysis of ultrasonic vocalisation does not allow chronic pain to be evaluated in rats. Pain 2002, 95, 165-173, doi:Doi 10.1016/S0304-3959(01)00394-3.

ee) Kurejova, M.; Nattenmuller, U.; Hildebrandt, U.; Selvaraj, D.; Stosser, S.; Kuner, R. An improved behavioural assay demonstrates that ultrasound vocalizations constitute a reliable indicator of chronic cancer pain and neuropathic pain. Mol Pain 2010, 6, 18, doi:10.1186/1744-8069-6-18.

ff) Niel, L.; Weary, D.M. Behavioural responses of rats to gradual-fill carbon dioxide euthanasia and reduced oxygen concentrations. Appl Anim Behav Sci 2006, 100, 295-308, doi:10.1016/j.applanim.2005.12.001.

gg) Sevcik, M.A.; Jonas, B.M.; Lindsay, T.H.; Halvorson, K.G.; Ghilardi, J.R.; Kuskowski, M.A.; Mukherjee, P.; Maggio, J.E.; Mantyh, P.W. Endogenous opioids inhibit early-stage pancreatic pain in a mouse model of pancreatic cancer. Gastroenterology 2006, 131, 900-910, doi:10.1053/j.gastro.2006.06.021.

hh) Williams, W.O.; Riskin, D.K.; Mott, A.K. Ultrasonic sound as an indicator of acute pain in laboratory mice. Journal of the American Association for Laboratory Animal Science : JAALAS 2008, 47, 8-10.

ii) Dalla Costa, E.; Pascuzzo, R.; Leach, M.C.; Dai, F.; Lebelt, D.; Vantini, S.; Minero, M. Can grimace scales estimate the pain status in horses and mice? A statistical approach to identify a classifier. PloS one 2018, 13, e0200339, doi:10.1371/journal.pone.0200339.

jj) Faller, K.M.E.; McAndrew, D.J.; Schneider, J.E.; Lygate, C.A. Refinement of analgesia following thoracotomy and experimental myocardial infarction using the Mouse Grimace Scale. Experimental Physiology 2015, 100, 164-172, doi:10.1113/expphysiol.2014.083139.

kk) Leach, M.C.; Klaus, K.; Miller, A.L.; Scotto di Perrotolo, M.; Sotocinal, S.G.; Flecknell, P.A. The assessment of post-vasectomy pain in mice using behaviour and the Mouse Grimace Scale. PloS one 2012, 7, e35656, doi:10.1371/journal.pone.0035656.

ll) Miller, A.L.; Golledge, H.D.; Leach, M.C. The Influence of Isoflurane Anaesthesia on the Rat Grimace Scale. PloS one 2016, 11, e0166652, doi:10.1371/journal.pone.0166652.

mm) Miller, A.L.; Leach, M.C. Using the mouse grimace scale to assess pain associated with routine ear notching and the effect of analgesia in laboratory mice. Lab Anim 2015, 49, 117-120, doi:10.1177/0023677214559084.

nn) Miller, A.L.; Leach, M.C. The effect of handling method on the mouse grimace scale in two strains of laboratory mice. Lab Anim 2016, 50, 305-307, doi:10.1177/0023677215622144.

oo) Seiffert, I.; van Dijk, R.M.; Koska, I.; Di Liberto, V.; Moller, C.; Palme, R.; Hellweg, R.; Potschka, H. Toward evidence-based severity assessment in rat models with repeated

seizures: III. Electrical post-status epilepticus model. Epilepsia 2019, 60, 1539-1551, doi:10.1111/epi.16095.

pp) Finlayson, K.; Lampe, J.F.; Hintze, S.; Wurbel, H.; Melotti, L. Facial Indicators of Positive Emotions in Rats. PloS one 2016, 11, doi:ARTN e0166446 10.1371/journal.pone.0166446.

qq) Beninson, J.A.; Lofgren, J.L.; Lester, P.A.; Hileman, M.M.; Berkowitz, D.J.; Myers, D.D., Jr. Analgesic Efficacy and Hematologic Effects of Robenacoxib in Mice. Journal of the American Association for Laboratory Animal Science : JAALAS 2018, 57, 258-267.

rr) Oliver, V.L.; Thurston, S.E.; Lofgren, J.L. Using Cageside Measures to Evaluate Analgesic Efficacy in Mice (Mus musculus) after Surgery. Journal of the American Association for Laboratory Animal Science : JAALAS 2018, 57, 186-201.

ss) Hager, C.; Keubler, L.M.; Biernot, S.; Dietrich, J.; Buchheister, S.; Buettner, M.; Bleich, A. Time to Integrate to Nest Test Evaluation in a Mouse DSS-Colitis Model. PloS one 2015, 10, doi:ARTN e014382410.1371/journal.pone.0143824.

tt) Jirkof, P. Burrowing and nest building behavior as indicators of well-being in mice. Journal of Neuroscience Methods 2014, 234, 139-146, doi:10.1016/j.jneumeth.2014.02.001.

uu) Jirkof, P.; Durst, M.; Klopfleisch, R.; Palme, R.; Thone-Reineke, C.; Buttgereit, F.; Schmidt-Bleek, K.; Lang, A. Administration of Tramadol or Buprenorphine via the drinking water for post-operative analgesia in a mouse-osteotomy model. Scientific reports 2019, 9, doi:ARTN 1074910.1038/s41598-019-47186-5.

vv) Deacon, R.M.J. Burrowing in rodents: a sensitive method for detecting behavioral dysfunction. Nature Protocols 2006, 1, 118-121, doi:10.1038/nprot.2006.19.

ww) Gjendal, K.; Ottesen, J.L.; Olsson, I.A.S.; Sorensen, D.B. Burrowing and nest building activity in mice after exposure to grid floor, isoflurane or ip injections. Physiol Behav 2019, 206, 59-66, doi:10.1016/j.physbeh.2019.02.022.

xx) Gould, S.A.; Doods, H.; Lamla, T.; Pekcec, A. Pharmacological characterization of intraplantar Complete Freund's Adjuvant-induced burrowing deficits. Behavioural brain research 2016, 301, 142-151, doi:10.1016/j.bbr.2015.12.019.

yy) Jirkof, P.; Cesarovic, N.; Rettich, A.; Nicholls, F.; Seifert, B.; Arras, M. Burrowing behavior as an indicator of post-laparotomy pain in mice. Frontiers in Behavioral Neuroscience 2010, 4, doi:ARTN 16510.3389/fnbeh.2010.00165.

zz) Rutten, K.; Robens, A.; Read, S.J.; Christoph, T. Pharmacological validation of a refined burrowing paradigm for prediction of analgesic efficacy in a rat model of sub-chronic knee joint inflammation. Eur J Pain 2014, 18, 213-222, doi:10.1002/j.1532-2149.2013.00359.x.

aaa) Whittaker, A.L.; Lymn, K.A.; Nicholson, A.; Howarth, G.S. The assessment of general well-being using spontaneous burrowing behaviour in a short-term model of

chemotherapy-induced mucositis in the rat. Laboratory Animals 2015, 49, 30-39, doi:10.1177/0023677214546913.

bbb) Jirkof, P.; Cesarovic, N.; Rettich, A.; Arras, M. Housing of female mice in a new environment and its influence on post-surgical behaviour and recovery. Appl Anim Behav Sci 2013, 148, 209-217, doi:10.1016/j.applanim.2013.08.006.

ccc) Jirkof, P.; Cesarovic, N.; Rettich, A.; Fleischmann, T.; Arras, M. Individual housing of female mice: influence on postsurgical behaviour and recovery. Laboratory Animals 2012, 46, 325-334, doi:10.1258/la.2012.012027.

ddd) Baumann, A.; Moreira, C.G.; Morawska, M.M.; Masneuf, S.; Baumann, C.R.; Noain, D. Preliminary Evidence of Apathetic-Like Behavior in Aged Vesicular Monoamine Transporter 2 Deficient Mice. Front Hum Neurosci 2016, 10, 587, doi:10.3389/fnhum.2016.00587.

eee) Jorgensen, B.P.; Hansen, J.T.; Krych, L.; Larsen, C.; Klein, A.B.; Nielsen, D.S.; Josefsen, K.; Hansen, A.K.; Sorensen, D.B. A Possible Link between Food and Mood: Dietary Impact on Gut Microbiota and Behavior in BALB/c Mice. PloS one 2014, 9, doi:ARTN e10339810.1371/journal.pone.0103398.

fff) Strekalova, T.; Steinbusch, H.W. Measuring behavior in mice with chronic stress depression paradigm. Prog Neuropsychopharmacol Biol Psychiatry 2010, 34, 348-361, doi:10.1016/j.pnpbp.2009.12.014.

ggg) van den Berg, R.; Laman, J.D.; van Meurs, M.; Hintzen, R.Q.; Hoogenraad, C.C. Rotarod motor performance and advanced spinal cord lesion image analysis refine assessment of neurodegeneration in experimental autoimmune encephalomyelitis. J Neurosci Methods 2016, 262, 66-76, doi:10.1016/j.jneumeth.2016.01.013.

hhh) Richter, S.H.; Kästner, N.; Kriwet, M.; Kaiser, S.; Sachser, N. Play matters: the surprising relationship between juvenile playfulness and anxiety in later life. Animal Behaviour 2016, 114, 261-271, doi:10.1016/J.ANBEHAV.2016.02.003.

iii) Kloke, V.; Heiming, R.S.; Bölting, S.; Kaiser, S.; Lewejohann, L.; Lesch, K.-P.; Sachser, N. Unexpected effects of early-life adversity and social enrichment on the anxiety profile of mice varying in serotonin transporter genotype. Behavioural brain research 2013, 247, doi:10.1016/j.bbr.2013.03.039.

jjj) Bert, B.; Schmidt, N.; Voigt, J.P.; Fink, H.; Rex, A. Evaluation of cage leaving behaviour in rats as a free choice paradigm. J Pharmacol Toxicol Methods 2013, 68, 240-249, doi:10.1016/j.vascn.2013.01.001.

kkk) Clement, Y.; Joubert, C.; Kopp, C.; Lepicard, E.M.; Venault, P.; Misslin, R.; Cadot, M.; Chapouthier, G. Anxiety in mice: a principal component analysis study. Neural plasticity 2007, 2007, 35457, doi:10.1155/2007/35457.

lll) Sahin, C.; Doostdar, N.; Neill, J.C. Towards the development of improved tests for negative symptoms of schizophrenia in a validated animal model. Behavioural brain research 2016, 312, 93-101, doi:10.1016/j.bbr.2016.06.021.

mmm) Dunbar, M.L.; David, E.M.; Aline, M.R.; Lofgren, J.L. Validation of a Behavioral Ethogram for Assessing Postoperative Pain in Guinea Pigs (Cavia porcellus). Journal of the American Association for Laboratory Animal Science : JAALAS 2016, 55, 29-34.

nnn) Paster, E.V.; Villines, K.A.; Hickman, D.L. Endpoints for mouse abdominal tumor models: refinement of current criteria. Comp Med 2009, 59, 234-241.

ooo) Pinkernell, S.; Becker, K.; Lindauer, U. Severity assessment and scoring for neurosurgical models in rodents. Laboratory Animals 2016, 50, 442-452, doi:10.1177/0023677216675010.

ppp) Spangenberg, E.M.; Keeling, L.J. Assessing the welfare of laboratory mice in their home environment using animal-based measures--a benchmarking tool. Lab Anim 2016, 50, 30-38, doi:10.1177/0023677215577298.

qqq) Golledge, H.; Jirkof, P. Score sheets and analgesia. Laboratory Animals 2016, 50, 411-413, doi:10.1177/0023677216675387.
